# Trends and Projections of Burden of Ischemic Heart Disease in China Versus Other G20 Countries: A Comparative Study Based on the 2021 Global Burden of Disease Database

**DOI:** 10.5334/gh.1424

**Published:** 2025-04-03

**Authors:** Yi Zhang, Hui Li, JingHan Chu, ShuaiShuai Ye, Chun Xiao, BuChun Zhang

**Affiliations:** 1 Department of Cardiology, The First Affiliated Hospital of USTC, Division of Life Sciences and Medicine, University of Science and Technology of China, Anhui Hefei, 230001, China; 2 Graduate School, Wannan Medical College, Anhui Wuhu, 241002, China; 3 School of Medical Technology, Tianjin University of Traditional Chinese Medicine, Tianjin 301617, China; 4 Department of Cardiology, Third People’s Hospital of Huizhou, Guangzhou Medical University, Guangdong 516002, China

**Keywords:** Ischemic heart disease, Global burden of disease, Risk factor, Temporal trends

## Abstract

**Objective:**

This study aims to analyse the burden of ischemic heart disease (IHD) in China and other G20 countries from 1990–2021 and predict the burden for the next decade.

**Methods:**

Using data from the Global Burden of Disease (GBD) 2021 study, we evaluated the age-standardised rates (ASRs) of incidence, prevalence, mortality and disability-adjusted life years (DALYs) by estimated annual percentage change (EAPC). The Bayesian age-period-cohort (BAPC) model was used to forecast the incidence, mortality and DALY rates of IHD in China from 2021–2040.

**Results:**

The ASRs of incidence, mortality and DALYs of IHD in China increased with EAPCs of 0.66 (95% CI: 0.50, 0.82), 0.97 (95% CI: 0.63, 1.31) and 0.51 (95% CI: 0.24, 0.78), respectively. Compared with other G20 countries, China was ranked 14th for the ASR of incidence in 1990 and then rose to 7th in 2021. The ASR of prevalence for IHD in China jumped from 8th in 1990 to 5th in 2021, and both the ASR of mortality and DALYs for IHD in China ranked 7th in 2021. The top five risk factors affecting mortality in China in 2021 were high systolic blood pressure, dietary risk, air pollution, high LDL cholesterol and tobacco. Over the next 20 years, the ASR of incidence, mortality and DALYs for IHD will increase continuously in males.

**Conclusion:**

The burden of IHD is expected to increase steadily in China, highlighting the urgency for early monitoring and preventative strategies, particularly focusing on the elderly and male populations.

## Introduction

Ischemic heart disease (IHD) is one of the most common cardiovascular diseases and a major threat to public health worldwide. IHD includes acute myocardial infarction, chronic stable angina, chronic IHD and associated heart failure (1). Over the past three decades, age-standardised incidence and mortality rates of IHD have either remained stable or declined in countries with a high Socio-Demographic Index (SDI), such as the United States and Australia (2). However, there have been large increases in IHD incidence rates in low and middle SDI countries (3). It should be noted that China has become the country with the highest number of new cases and deaths from IHD each year. A previous study showed an increasing trend in the incidence of IHD in China from 1990–2019, with the most significant increases in the age-standardised mortality rate (ASMR) and age-standardised disability-adjusted life year (DALY) rate (ASDR) (4).

The reasons for the rapidly increasing trends in IHD incidence and mortality are unclear, but are likely to be influenced by a combination of modifiable and non-modifiable risk factors such as insulin resistance, hypertension, diabetes mellitus, dyslipidaemia and physical inactivity (5,6). However, it is not well understood whether the known risk factors play similar roles in contributing to the burden of IHD in China as in other G20 countries. Identifying risk factors is also essential towards optimising guidelines for preventing and detecting IHD.

The Group of Twenty (G20) is an international forum for economic cooperation that includes a mix of developed and developing countries representing nearly two-thirds of the world’s population (7). The burden of disease in the G20 countries is inextricably linked to their economic environment and health status (8). Previous studies have shown that the burden of IHD varies according to geographical location and socio-economic status (9,10). However, a comprehensive analysis of the burden of disease, long-term trends and attributable risk factors for IHD in China and other G20 member countries is not available. A better understanding of the current state and future trends in disease burden can promote effective prevention and help policy-makers to allocate health resources accurately and efficiently. Therefore, in this study, we used data from GBD 2021 to quantify the trends in incidence, prevalence, mortality and DALYs of IHD in China and compared these indicators with other G20 countries from 1990–2021, as well as the contributions of risk factors. We also projected the burden in China to 2040.

## Methods

### Study design and data sources

This study is a secondary analysis based on the 2021 GBD dataset, a comprehensive database covering incidence, prevalence and mortality of more than 371 diseases and injuries in 204 countries and regions. The GBD dataset was launched in 1990 with the aim of providing timely, valid and relevant assessments of critical health outcomes. Detailed methods for GBD 2021 have been previously documented (11). For this study, the following annual information was retrieved from 1990–2021: GBD estimate (cases of death or injury), measure (prevalence, incidence, deaths, DALYs), metric (rate, number), location (G20 countries including Argentina, Australia, Brazil, Canada, China, European Union, France, Germany, India, Indonesia, Italy, Japan, Mexico, Republic of Korea, Russian Federation, Saudi Arabia, South Africa, Turkey, United Kingdom and United States of America), age (all ages, age-standardised, age groups >25–94 years at 5-year intervals), sex (both, female, male), year (between 1990–2021).

### Case definition

IHD is also known as coronary artery disease. IHD includes acute myocardial infarction (MI) and chronic IHD (angina and asymptomatic IHD following MI) (12). MI is diagnosed according to the third edition of the global definition of MI, and it is included in out-of-hospital sudden cardiac death. A diagnosis of angina pectoris can be made using the Rose Angina Questionnaire, a definitive diagnosis by a physician, or medical documentation of the use of nitrate medications to relieve chest pain (13). In GBD 2021, IHD was defined in accordance with the 10th revision of the ICD (codes I20–I21.6).

### Data analysis

The study retrieved disease burden metrics with 95% uncertainty intervals (UIs), including absolute numbers and age-standardised rates (ASR, per 100,000 population) of prevalence, incidence, mortality and DALYs, presented as age-standardised prevalence rate (ASPR), age-standardised incidence rate (ASIR), ASMR and ASDR. In addition, the estimated annual percentage change (EAPC) was calculated to assess the time trends in the burden of IHD during the period 1990–2021. The EAPC is a widely accepted measure to quantify the trend in age-standardised rates over specific time intervals and was calculated based on the regression model fitted to the natural logarithm of the rates (14). The 95% confidence interval (CI) was also determined by the linear regression model. If the 95% CI of the corresponding EAPC estimate is >0, the age-standardized indicator shows an increasing trend; if <0, it shows a decreasing trend; if it includes 0, it shows a stable trend. The Bayesian Age-Period-Cohort (BAPC) model is a complex statistical tool that combines prior information about unknown parameters with sample information to estimate the posterior distribution and infer these unknown parameters (15). It has been shown to be more accurate in predicting disease burden. Therefore, we used it in the integrated nested Laplace approximation (INLA) using the R packages BAPC and INLA to predict the ASIR, ASMR and ASDR of IHD in China from 2021–2040. All statistical analyses and data visualizations were performed using R (version 4.3.3) and JD_GBDR (V2.24, Jingding Medical Technology Co., Ltd.). *P* value <0.05 was considered to be statistically significant.

### Ethics

The institutional review board granted an exemption for this study, as it utilized publicly accessible data that contained no confidential or personally identifiable patient information.

### Informed consent

Informed consent was not needed.

## Results

### Overall trend of ischemic heart disease in China and other G20 countries

In China, there were 2,301,643.54 (95% UI: 1, 861,968.54–2,792,193.37) incident cases of IHD in 1990 and 7,304,573.22 (95% UI: 5,815,313.24–8,949,994.68) cases in 2021, an increase of 217.36% from 1990–2021. China’s population has grown over the past three decades from around 1.13 billion in 1990 to 1.41 billion in 2021, according to the World Bank Group. Meanwhile, the global incidence of IHD will be 31,872,778.18 (95% UI: 26,284,920.94–38,267,834.30) cases in 2021, increasing from 15,813,618.65 (95% UI: 13,180,529.44–18,849,478.54) cases in 1990. The ASIR of IHD in China were 315.31 (95% UI: 255.53–382.49) in 1990 and 365.67 (95% UI: 293.32–440.07) in 2021 per 100,000 population, which were lower than the global average of 419.54 (95% UI: 351.07–498.15) in 1990 and 372.90 (95% UI: 307.95–444.19) in 2021 per 100,000 population. When compared to G20 countries, China was ranked 14th in the ASIR in 1990 and then increased to 7th in 2021 (Table 1). IHD accounted for 19,505,463.06 (95% UI: 16,754,811.28–22,537,174.00) prevalent cases in 1990 and 63,331,311.50 (95% UI: 53,812,323.83–76,196,537.00) cases in 2021 in China, comprising 0.17% of global prevalent cases in 1990 and 24.91% in 2021. The ASPR of IHD in China jumped from 8th in 1990 to 5th in 2021 (Table 2). Overall, the number of IHD mortality cases in China increased from 547,845.09 in 1990 to 1,956,859.43 in 2021, an increase of 257.19%. During the same period, the ASMR in China increased significantly from 94.14 (95% UI: 84.01–105.89) in 1990 to 110.91 (95% UI: 92.42–128.56) in 2021. Interestingly, the G20 countries showed a decrease in ASMR from 1990–2021, with the exception of India and Indonesia (Table 3). Similarly, the DALY increased by 161.83% from 13,624,111.86 in 1990 to 35,672,627.04 in 2021. The ASDR in China in 1990 was 1,771.13 (95%UI 1,574.76–1,990.67), but in 2019 China’s ASDR increased to 1,856.51 (95%UI 1,548.73–2,159.82), showing an upward trend compared with most of the G20 countries (Table 4).

**Table 1:** Incidence of ischemic heart disease in G20 countries and temporal trends from 1990 to 2021.


LOCATION	CASES (95% UI)			AGE-STANDARDIZED INCIDENCE RATE, PER 100 000 (95% UI)		
	
	1990	2021	1990–2021 CHANGE (%)	1990	2021	1990–2021 EAPC(95% *CI*)

Global	15813618.65 (13180529.44, 18849478.54)	31872778.18 (26284920.94, 38267834.30)	101.55 (98.87, 104.39)	419.54 (351.07, 498.15)	372.90 (307.95, 444.19)	–0.44 (–0.47, –0.41)

China	2301643.54 (1861968.54, 2792193.37)	7304573.22 (5815313.24, 8949994.68)	217.36 (202.99, 235.54)	315.31 (255.53, 382.49)	365.67 (293.32, 440.07)	0.66 (0.50, 0.82)

Argentina	110811.76 (97236.50, 128208.93)	135407.29 (115179.33, 160520.07)	22.20 (13.90, 31.04)	357.69 (315.71, 410.38)	243.87 (207.22, 291.57)	–1.61 (–1.89, –1.33)

Australia	65818.16 (58075.69, 74535.04)	94563.21 (77489.08, 114340.96)	43.67 (28.11, 61.46)	344.16 (305.95, 389.10)	213.72 (176.25, 262.25)	–1.43 (–1.82, –1.05)

Brazil	176403.78 (147670.15, 209651.27)	420771.58 (341548.72, 505774.15)	138.53 (127.34, 149.74)	197.12 (163.41, 234.79)	167.57 (136.27, 200.40)	–0.37 (–0.45, –0.29)

Canada	128768.69 (113261.44, 149684.38)	146396.51 (123174.69, 174147.54)	13.69 (4.32, 25.89)	397.51 (349.10, 463.63)	204.24 (170.67, 245.09)	–2.65 (–2.90, –2.40)

European Union	2093991.53 (1890015.76, 2353146.47)	1981694.26 (1734015.05, 2269780.24)	–5.36 (–9.49, –1.25)	352.76 (317.87, 392.91)	207.49 (179.16, 242.53)	–1.90 (–2.03, –1.77)

France	233422.28 (209001.93, 261151.14)	249807.82 (214444.42, 294830.86)	7.02 (–4.44, 20.56)	283.69 (256.56, 314.88)	176.27 (147.06, 213.19)	–0.98 (–1.20, –0.77)

Germany	551176.49 (505918.16, 601471.06)	441721.75 (391901.28, 505338.72)	–19.86 (–28.10, –9.94)	432.87 (399.23, 469.58)	223.88 (195.43, 261.15)	–2.34 (–2.45, –2.24)

India	2704532.05 (2148026.31, 3347899.60)	6849890.32 (5528403.36, 8331623.25)	153.27 (145.59, 161.95)	607.77 (484.82, 748.75)	585.23 (470.56, 713.17)	–0.14 (–0.23, –0.06)

Indonesia	211687.37 (168424.18, 260333.64)	582119.57 (463533.37, 718983.05)	174.99 (168.63, 180.91)	240.21 (192.05, 295.20)	270.65 (215.27, 331.46)	0.61 (0.50, 0.71)

Italy	227442.28 (178385.67, 281938.49)	226379.46 (187770.03, 263972.40)	–0.47 (–8.76, 9.97)	262.22 (206.08, 325.30)	166.39 (138.51, 194.88)	–1.71 (–1.81, –1.62)

Japan	150408.68 (118335.29, 188174.43)	358364.99 (279728.98, 453220.78)	138.26 (111.67, 168.37)	91.85 (72.89, 114.39)	90.63 (71.46, 113.97)	–0.30 (–0.47, –0.12)

Mexico	146333.26 (117620.58, 178933.75)	412013.58 (329516.20, 507005.80)	181.56 (174.10, 190.11)	350.94 (278.60, 435.25)	329.42 (261.01, 403.11)	–0.30 (–0.37, –0.24)

Republic of Korea	50995.36 (43011.51, 60717.02)	88757.79 (73096.64, 107551.37)	74.05 (55.76, 94.14)	207.36 (179.12, 241.59)	96.28 (79.26, 116.32)	–2.18 (–2.59, –1.77)

Russian Federation	1226018.72 (986689.42, 1484619.58)	1719384.92 (1372804.25, 2108619.52)	40.24 (31.88, 48.06)	741.29 (599.61, 898.59)	727.97 (586.20, 880.94)	–0.35 (–0.66, –0.03)

Saudi Arabia	50790.91 (41793.15, 61699.52)	202757.82 (167092.42, 247500.47)	299.20 (266.89, 340.58)	877.87 (719.09, 1065.04)	920.14 (777.97, 1085.34)	0.16 (0.04, 0.28)

South Africa	85209.09 (67282.25, 106271.37)	172647.18 (136318.87, 217054.32)	102.62 (96.61, 108.07)	419.96 (329.42, 528.11)	380.13 (300.49, 471.61)	–0.52 (–0.62, –0.43)

Turkey	287173.65 (248897.84, 339689.62)	621078.92 (527076.81, 740295.19)	116.27 (95.59, 138.03)	824.26 (718.28, 963.21)	665.07 (568.25, 784.58)	–0.75 (–0.87, –0.64)

United Kingdom	258729.50 (208292.78, 318199.59)	176843.92 (143926.41, 213528.89)	–31.65 (–35.87, –26.82)	287.52 (232.83, 354.16)	141.62 (115.22, 170.01)	–2.12 (–2.49, –1.76)

United States of America	1452572.29 (1131039.12, 1825893.46)	967989.81 (814312.97, 1134211.96)	–33.36 (–39.52, –25.08)	461.63 (361.24, 582.38)	170.36 (143.90, 197.67)	–3.66 (–3.95, –3.37)


G20: Group of 20, UI: Uncertainty interval, EAPC: Estimated annual percentage change, CI: Confidence interval.

**Table 2:** Prevalence of ischemic heart disease in G20 countries and temporal trends from 1990 to 2021.


LOCATION	CASES (95% UI)			AGE-STANDARDIZED PREVALENCE RATE, PER 100 000 (95% UI)		
	
	1990	2021	1990–2021 CHANGE (%)	1990	2021	1990–2021 EAPC (95% *CI*)

Global	112169488.40 (99416740.98, 125730168.70)	254276267.90 (221446458.10, 295493092.70)	126.69 (117.83, 136.62)	2904.72 (2575.99, 3248.10)	2946.38 (2572.69, 3424.32)	0.00 (–0.03, 0.03)

China	19505463.06 (16754811.28, 22537174.00)	63331311.50 (53812323.83, 76196537.00)	224.68 (208.12, 245.47)	2526.44 (2189.97, 2914.97)	3042.35 (2601.68, 3629.87)	0.64 (0.56, 0.72)

Argentina	638248.00 (583732.87, 701214.99)	932786.44 (837288.33, 1049343.95)	46.15 (37.57, 54.96)	1977.98 (1811.70, 2166.45)	1666.56 (1499.65, 1871.35)	–0.73 (–0.83, –0.64)

Australia	438617.93 (407006.55, 476441.50)	847760.59 (760154.86, 952136.02)	93.28 (76.44, 108.90)	2247.86 (2092.46, 2437.69)	1877.69 (1685.68, 2103.19)	–0.65 (–0.85, –0.44)

Brazil	1724721.45 (1475061.00, 1976421.82)	4966552.33 (4177586.92, 5843929.62)	187.96 (175.62, 201.95)	1954.18 (1672.80, 2253.55)	1976.74 (1669.26, 2325.71)	0.07 (0.04, 0.10)

Canada	742086.71 (684174.01, 806721.39)	1105820.17 (1000313.19, 1252450.95)	49.01 (39.60, 58.73)	2267.31 (2093.18, 2463.98)	1544.09 (1400.94, 1734.92)	–1.48 (–1.63, –1.34)

European Union	14156038.48 (12731179.84, 15687865.33)	17292412.64 (15335809.23, 19574701.89)	22.16 (17.24, 28.03)	2348.86 (2116.10, 2596.35)	1855.83 (1644.65, 2094.96)	–0.93(–1.01, –0.86)

France	1614378.54 (1467211.54, 1788197.60)	2180522.62 (1942324.60, 2473239.35)	35.07 (25.31, 45.93)	1959.02 (1790.62, 2161.32)	1595.12 (1425.27, 1801.50)	–0.78 (–0.88, –0.68)

Germany	3101107.62 (2833708.93, 3409588.35)	3151797.82 (2791317.00, 3549641.44)	1.63 (–4.26, 8.69)	2442.44 (2243.92, 2676.81)	1683.17 (1496.00, 1900.56)	–1.28 (–1.34, –1.21)

India	17867820.10 (15107259.55, 20818190.22)	51933273.32 (43141707.52, 62567272.58)	190.65 (176.16, 207.47)	4110.85 (3518.51, 4788.48)	4458.18 (3744.48, 5412.76)	0.27 (0.25, 0.28)

Indonesia	1842025.57 (1574670.90, 2126112.82)	5158083.01 (4325307.05, 6144843.95)	180.02 (166.62, 193.01)	2085.58 (1796.26, 2404.55)	2349.89 (1998.83, 2782.38)	0.50 (0.46, 0.54)

Italy	1682045.64 (1429035.51, 1944088.91)	2109380.70 (1762593.05, 2505110.62)	25.41 (19.46, 31.57)	1887.10 (1615.66, 2179.93)	1544.57 (1296.30, 1834.07)	–0.88 (–0.97, –0.80)

Japan	1590718.02 (1362924.60, 1845351.10)	2918517.07 (2466761.27, 3474093.52)	83.47 (66.73, 100.04)	943.84 (811.03, 1090.14)	802.34 (686.82, 938.16)	–0.77 (–0.88, –0.67)

Mexico	1163203.38 (1006940.36, 1337844.20)	3513288.55 (2974794.29, 4204715.90)	202.04 (188.56, 217.47)	2798.08 (2417.87, 3240.52)	2797.04 (2384.64, 3344.04)	–0.08 (–0.12, –0.03)

Republic of Korea	304843.32 (279161.93, 336172.44)	788589.59 (697856.86, 892037.47)	158.69 (142.00, 176.90)	1118.13 (1028.29, 1232.87)	841.83 (748.12, 948.82)	–1.12 (–1.25, –0.99)

Russian Federation	8242544.07 (7192086.29, 9399669.62)	12624157.82 (10898100.28, 14905229.20)	53.16 (46.01, 60.62)	4758.48 (4182.91, 5385.01)	5279.14 (4568.46, 6189.43)	0.23 (0.12, 0.34)

Saudi Arabia	344962.97 (321903.79, 370003.13)	1407289.37 (1272028.51, 1545986.29)	307.95 (285.53, 332.54)	6267.77 (5855.70, 6718.94)	7341.64 (6747.53, 7966.80)	0.59 (0.54, 0.64)

South Africa	599803.54 (513181.53, 695631.29)	1283629.91 (1076863.48, 1532287.96)	114.01 (104.31, 124.61)	3009.73 (2580.12, 3500.90)	2839.86 (2396.41, 3389.41)	–0.31 (–0.38, –0.24)

Turkey	2046513.35 (1921458.97, 2196549.85)	5015695.28 (4562044.65, 5507294.31)	145.08 (129.89, 159.57)	6090.46 (5727.54, 6507.62)	5365.86 (4895.52, 5874.65)	–0.55 (–0.61, –0.50)

United Kingdom	1755046.17 (1523281.49, 2014513.46)	1702773.46 (1453038.01, 2001056.49)	–2.98 (–7.44, 2.33)	1953.27 (1692.63, 2228.79)	1363.38 (1162.34, 1599.93)	–1.45 (–1.63, –1.27)

United States of America	9341215.83 (7880209.71, 10980078.81)	8655573.75 (7305087.14, 10307536.66)	–7.34 (–11.90, –1.84)	2912.81 (2469.35, 3410.96)	1488.78 (1263.92, 1756.36)	–2.57 (–2.76, –2.38)


G20: Group of 20, UI: Uncertainty interval, EAPC: Estimated annual percentage change, CI: Confidence interval.

**Table 3:** Mortality of ischemic heart disease in G20 countries and temporal trends from 1990 to 2021.


LOCATION	CASES (95% UI)			AGE-STANDARDIZED MORTALITY RATE, PER 100 000 (95% UI)		
	
	1990	2021	1990–2021 CHANGE (%)	1990	2021	1990–2021 EAPC (95% *CI*)

Global	5367136.58 (5076403.86, 5562773.87)	8991636.68 (8264123.21, 9531130.17)	67.53 (58.76, 75.87)	158.90 (148.14, 165.30)	108.73 (99.60, 115.38)	–1.30 (–1.34, –1.26)

China	547845.09 (486106.46, 617005.67)	1956859.43 (1634477.57, 2280131.16)	257.19 (196.40, 335.87)	94.14 (84.01, 105.89)	110.91 (92.42, 128.56)	0.97 (0.63, 1.31)

Argentina	46663.19 (44422.94, 48177.52)	35109.80 (32241.92, 37063.90)	–24.76 (–28.75, –21.25)	158.80 (149.31, 164.64)	60.79 (55.95, 64.11)	–2.68 (–2.87, –2.50)

Australia	32990.15 (30571.98, 34270.32)	23031.87 (19557.67, 24978.12)	–30.19 (–36.36, –26.04)	175.07 (160.93, 182.55)	44.08 (37.95, 47.53)	–4.68 (–4.80, –4.56)

Brazil	105307.12 (100047.13, 108323.43)	157550.11 (144833.10, 165499.38)	49.61 (42.63, 56.02)	136.61 (127.24, 141.52)	64.01 (58.63, 67.35)	–2.32 (–2.41, –2.22)

Canada	50105.55 (46274.45, 51988.65)	41493.63 (36498.59, 44418.75)	–17.19 (–22.65, –13.96)	156.66 (143.99, 162.88)	51.12 (45.43, 54.44)	–3.88 (–4.04, –3.72)

European Union	1010274.38 (942751.82, 1041504.02)	744989.27 (653289.45, 794950.53)	–26.26 (–31.20, –23.24)	168.750 (156.699, 174.456)	64.652 (57.527, 68.531)	–3.303 (–3.387, –3.220)

France	74095.08 (68294.19, 77384.55)	56002.42 (47365.54, 60965.03)	–24.42 (–31.49, –20.09)	84.26 (77.70, 87.99)	29.87 (25.89, 32.30)	–3.48 (–3.61, –3.35)

Germany	254432.16 (233570.91, 265106.53)	150616.94 (129273.89, 163023.29)	–40.80 (–45.92, –37.72)	192.40 (176.05, 200.95)	63.67 (55.51, 68.32)	–3.72 (–3.84, –3.61)

India	571504.96 (511213.90, 624257.48)	1632881.84 (1487219.38, 1788197.88)	185.72 (150.37, 227.51)	137.87 (121.54, 151.08)	151.17 (137.46, 165.08)	0.48 (0.30, 0.66)

Indonesia	89011.81 (76060.38, 101756.30)	276493.65 (229238.18, 322155.06)	210.63 (148.12, 285.88)	102.96 (85.94, 119.91)	143.25 (119.34, 163.30)	1.19 (1.09, 1.29)

Italy	94745.72 (86023.99, 99143.84)	85030.62 (70074.59, 92946.70)	–10.25 (–18.86, –5.76)	108.50 (97.93, 113.85)	44.27 (37.56, 47.75)	–3.04 (–3.14, –2.94)

Japan	102221.70 (92558.19, 106893.75)	123164.00 (99194.64, 136240.43)	20.49 (6.95, 28.20)	66.69 (59.50, 70.22)	25.36 (21.70, 27.29)	–2.95 (–3.18, –2.72)

Mexico	38261.91 (36879.95, 38955.55)	129103.48 (114982.51, 143052.14)	237.42 (204.25, 271.86)	113.39 (108.31, 115.87)	113.21 (100.89, 125.20)	–0.10 (–0.37, 0.18)

Republic of Korea	12338.69 (10729.60, 14137.60)	25415.44 (20651.61, 29168.94)	105.98 (77.06, 138.93)	60.15 (51.83, 68.91)	28.27 (22.87, 32.57)	–3.03 (–3.31, –2.74)

Russian Federation	482850.75 (462864.86, 492436.80)	512524.27 (470911.59, 551237.97)	6.15 (–0.83, 13.27)	311.27 (294.21, 318.67)	212.91 (195.61, 229.02)	–1.61 (–2.12, –1.10)

Saudi Arabia	11469.22 (8755.15, 14415.79)	32329.67 (26303.88, 39773.79)	181.88 (105.51, 306.28)	225.52 (174.28, 279.62)	185.90 (158.13, 219.08)	–0.72 (–0.92, –0.51)

South Africa	13911.29 (11672.35, 15652.43)	30300.97 (27568.30, 32899.92)	117.82 (97.51, 147.85)	74.43 (61.29, 84.33)	78.04 (71.05, 84.66)	0.00 (–0.40, 0.41)

Turkey	61298.30 (54354.67, 68192.65)	112054.57 (92076.78, 131673.92)	82.80 (48.99, 119.78)	204.65 (180.71, 228.18)	133.38 (109.89, 155.94)	–1.29 (–1.60, –0.97)

United Kingdom	184357.71 (173164.20, 189356.76)	76354.24 (67535.95, 80433.70)	–58.58 (–61.06, –57.34)	196.49 (184.68, 201.92)	52.11 (46.86, 54.65)	–4.67 (–4.86, –4.48)

United States of America	594729.09 (534528.60, 623663.90)	493222.04 (432450.65, 527138.63)	–17.07 (–20.15, –14.93)	179.83 (162.05, 188.39)	78.92 (69.93, 83.85)	–3.01 (–3.16, –2.85)


G20: Group of 20, UI: Uncertainty interval, EAPC: Estimated annual percentage change, CI: Confidence interval.

**Table 4:** Disability-adjusted life years of ischemic heart disease in G20 countries and temporal trends from 1990 to 2021.


LOCATION	CASES (95% UI)			AGE-STANDARDIZED DALY RATE, PER 100 000 (95% UI)		
	
	1990	2021	1990–2021 CHANGE (%)	1990	2021	1990–2021 EAPC (95% *CI*)

Global	119162957.30 (114547786.90, 123454733.00)	188360557.30 (177036930.40, 198154476.60)	58.07 (49.50, 66.45)	3107.61 (2966.50, 3222.67)	2212.16 (2075.54, 2327.61)	–1.20 (–1.25, –1.15)

China	13624111.86 (12056605.55, 15466092.35)	35672627.04 (29920272.75, 41738945.76)	161.83 (112.90, 223.36)	1771.13 (1574.76, 1990.67)	1856.51 (1548.73, 2159.82)	0.51 (0.24, 0.78)

Argentina	951849.68 (921724.72, 978424.58)	659381.08 (620826.60, 690368.78)	–30.73 (–33.73, –27.66)	3045.51 (2935.24, 3135.27)	1179.87 (1113.64, 1234.14)	–2.74 (–2.91, –2.58)

Australia	612460.76 (578633.78, 632274.49)	356731.81 (318531.19, 379798.16)	–41.75 (–45.60, –38.77)	3168.21 (2983.34, 3273.46)	768.62 (696.10, 814.23)	–4.78 (–4.93, –4.63)

Brazil	2626269.58 (2536094.83, 2689422.79)	3713582.76 (3497727.16, 3865675.69)	41.40 (36.13, 46.96)	2916.95 (2791.89, 2996.12)	1469.72 (1380.14, 1530.27)	–2.16 (–2.24, –2.09)

Canada	929566.80 (881760.23, 956834.54)	673922.76 (612998.87, 710472.29)	–27.50 (–31.46, –24.85)	2886.93 (2735.29, 2972.96)	922.52 (849.26, 968.23)	–3.88 (–4.03, –3.73)

European Union	18823858.58 (18002268.61, 19273994.03)	11627757.69 (10603987.65, 12278527.32)	–38.23 (–41.68, –35.85)	3182.76 (3041.70, 3259.52)	1167.52 (1080.19, 1226.80)	–3.47 (–3.56, –3.38)

France	1265075.45 (1190602.88, 1315058.77)	841382.13 (745327.47, 909860.04)	–33.49 (–38.33, –29.80)	1519.29 (1434.08, 1575.77)	555.98 (503.45, 597.26)	–3.36 (–3.46, –3.25)

Germany	4427915.18 (4180700.20, 4575610.71)	2250722.06 (1988200.08, 2398480.24)	–49.17 (–52.95, –46.89)	3470.96 (3277.21, 3583.26)	1096.63 (993.52, 1160.04)	–3.86 (–3.98, –3.74)

India	16765036.26 (15156253.18, 18285253.38)	41316678.39 (37458752.56, 45305507.28)	146.45 (115.61, 184.29)	3321.51 (2980.30, 3628.90)	3400.03 (3098.47, 3720.83)	0.18 (0.06, 0.30)

Indonesia	2602187.39 (2270109.54, 2942989.62)	7347522.27 (6101893.28, 8695819.81)	182.36 (123.55, 249.41)	2420.31 (2079.08, 2751.00)	3043.08 (2527.28, 3544.71)	0.87 (0.77, 0.97)

Italy	1730943.85 (1627559.99, 1788916.04)	1194535.48 (1035883.32, 1282895.98)	–30.99 (–36.39, –28.01)	1988.69 (1868.54, 2056.66)	747.14 (673.73, 791.65)	–3.32 (–3.40, –3.25)

Japan	1848886.15 (1727631.33, 1913031.12)	1803042.97 (1556452.86, 1940530.38)	–2.48 (–10.13, 2.22)	1141.16 (1058.45, 1184.99)	502.25 (460.02, 526.29)	–2.51 (–2.66, –2.37)

Mexico	832032.19 (811517.00, 845792.79)	2626717.04 (2336523.15, 2939559.09)	215.70 (182.06, 250.77)	2050.57 (1986.67, 2089.13)	2125.69 (1892.12, 2373.05)	–0.03 (–0.30, 0.23)

Republic of Korea	297271.06 (258621.90, 336200.60)	424498.34 (362480.24, 475146.80)	42.80 (25.34, 62.76)	1084.12 (948.59, 1232.98)	470.99 (402.59, 526.79)	–3.26 (–3.52, –3.00)

Russian Federation	10020381.70 (9747362.61, 10188133.54)	9631666.32 (8865971.18, 10363140.76)	–3.88 (–10.54, 3.01)	5901.48 (5703.59, 6010.77)	4082.19 (3760.50, 4389.11)	–1.75 (–2.34, –1.15)

Saudi Arabia	308806.62 (233829.26, 392612.54)	1075688.88 (851631.30, 1338612.93)	248.34 (149.43, 412.45)	4888.39 (3749.14, 6143.97)	4219.77 (3530.63, 5082.93)	–0.42 (–0.65, –0.20)

South Africa	358547.87 (315697.05, 396157.61)	712623.05 (653012.88, 778831.81)	98.75 (80.86, 118.87)	1647.75 (1419.91, 1832.50)	1568.46 (1435.50, 1705.97)	–0.28 (–0.68, 0.13)

Turkey	1477735.15 (1299333.12, 1653583.77)	2187118.64 (1808029.13, 2591481.17)	48.00 (19.23, 80.44)	4255.11 (3773.12, 4724.64)	2419.28 (2008.07, 2856.99)	–1.92 (–2.16, –1.67)

United Kingdom	3430621.38 (3292789.25, 3498042.69)	1273352.28 (1174184.97, 1329582.17)	–62.88 (–64.42, –61.92)	3846.95 (3702.17, 3918.72)	983.93 (921.73, 1021.77)	–4.76 (–4.97, –4.56)

United States of America	10780285.70 (10063311.90, 11136811.70)	8751736.16 (8013896.50, 9182127.60)	–18.82 (–20.94, –16.94)	3395.99 (3185.06, 3501.20)	1527.33 (1412.25, 1595.15)	–2.90 (–3.05, –2.74)


G20: Group of 20, UI: Uncertainty interval, EAPC: Estimated annual percentage change, CI: Confidence interval, DALY, Disability-adjusted life years.

### Temporal trends for ischemic heart disease burden over time in China and other G20 countries

With the exception of China, Indonesia and Saudi Arabia, the ASIR for IHD decreased in all other G20 countries between 1990–2021. In China, the ASIR for IHD showed an increasing trend with an EAPC of 0.66 (95% CI: 0.50–0.82) (Table 1). In terms of ASPR for IHD during the period 1990–2021, Brazil, China, India, Indonesia, Saudi Arabia and the Russian Federation had increasing trends, whereas decreasing trends were observed in Australia, Argentina, Canada, France, Germany, Italy, Japan, South Africa, Turkey, the European Union, the United States of America and the United Kingdom (Table 2). The ASMR decreased in all G20 countries except China, India and Indonesia (Table 3). The ASDR of IHD increased in China (EAPC = 0.51, 95% CI: 0.24–0.78), and China’s upward trend ranked second in the G20 countries after Indonesia (Table 4).

### Age and sex distrubtion for ischemic heart disease burden in China and other G20 countries

**Figure 1:**
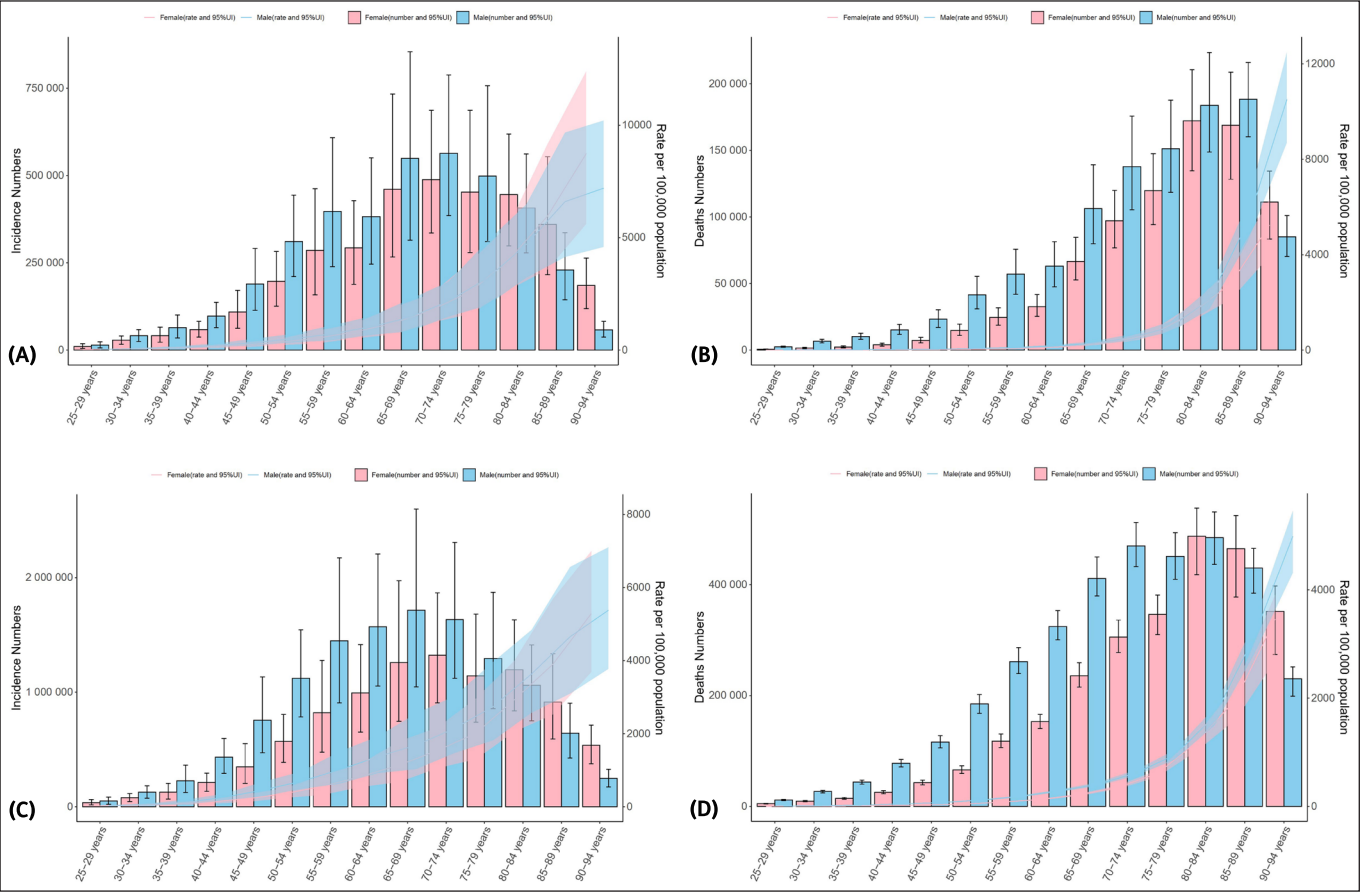
The age distribution for incidence and mortality of ischemic heart disease in China and G20 countries. **(A)** Age distribution of incidence in China for genders. **(B)** Age distribution of mortality in China for genders. **(C)** Age distribution of incidence in the G20 countries for genders. **(D)** Age distribution of mortality in the G20 countries for genders. G20: Group of Twenty.

Figure 1 illustrates the incidence and deaths numbers and rates for IHD in different age groups and genders in 2021. The incidence of IHD from China increases with age up to the 70–74 age group, where it peaks and then gradually declines. Both males and females exhibit this trend (Figure 1A). The number of mortality increased with age in both sexes and peaked in the 80–84 and 85–89 age groups (Figure 1B). For G20 countries, the highest number of incidence peaking at age subgroups 65–69 years and 70–74 years (Figure 1C). For both sexes, the highest number of mortality occurrs in the age subgroups 80–84 years (Figure 1D). From 1990–2021, the ASIR, ASPR, ASMR and ASDR in China show an upward trend compared to other G20 countries, with the over-80 age group showing the most significant upward trend (Supplementary Figure 1A–D).

### Risk factors for mortality of ischemic heart disease in China and other G20 countries

**Figure 2:**
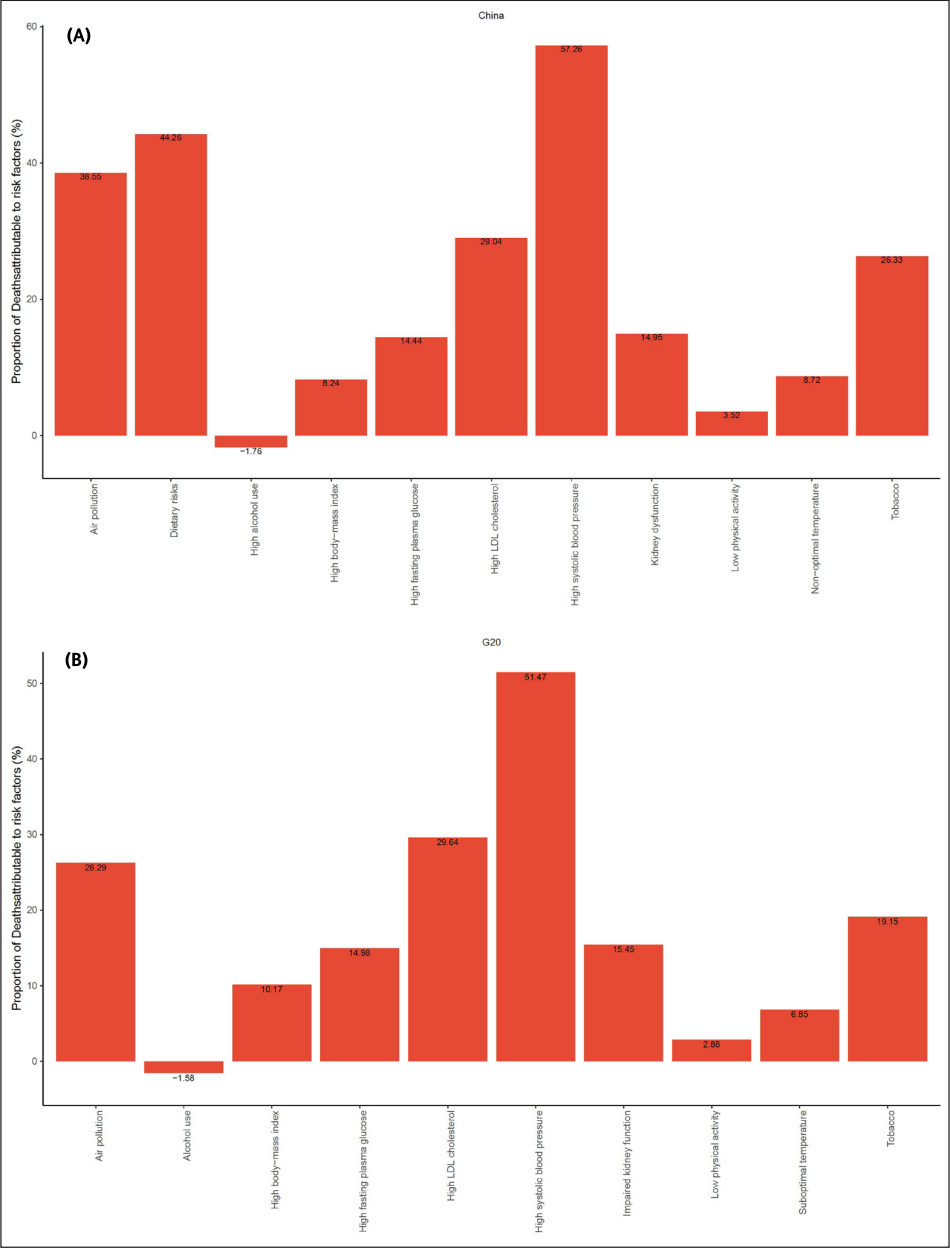
Attributable age-standardized mortality rates by ischemic heart disease risk factors in China and G20 countries in 2021. **(A)** China; **(B)** G20 countries. G20: Group of Twenty.

Although mortality from IHD in China was attributed to various individual risk factors, the top five risk factors in 2021 were high systolic blood pressure (57.26% (95% UI: 43.44–73.48)), dietary risks (44.26% (95% UI: 1.17–75.22)), air pollution (38.55%(95%UI:28.00–49.60)), high LDL cholesterol (29.04% (95%UI:18.00–42.02)), and tobacco (26.33% (95%UI: 19.84–33.55)) (Figure 2A). For G20 countries, the leading risk factors in 2021 were high systolic blood pressure (51.47% (95% UI: 40.95–61.05)), high LDL cholesterol (29.65% (95% UI: 19.83–40.06)), air pollution (26.29% (95% UI: 19.49–33.17)), tobacco (19.15% (95% UI: 15.75–22.79)), and impaired kidney function (15.45% (95% UI: 10.56–19.85)) (Figure 2B).

### Predictions of incidence, mortality and DALYs of ischemic heart disease in China from 2020 to 2040

**Figure 3:**
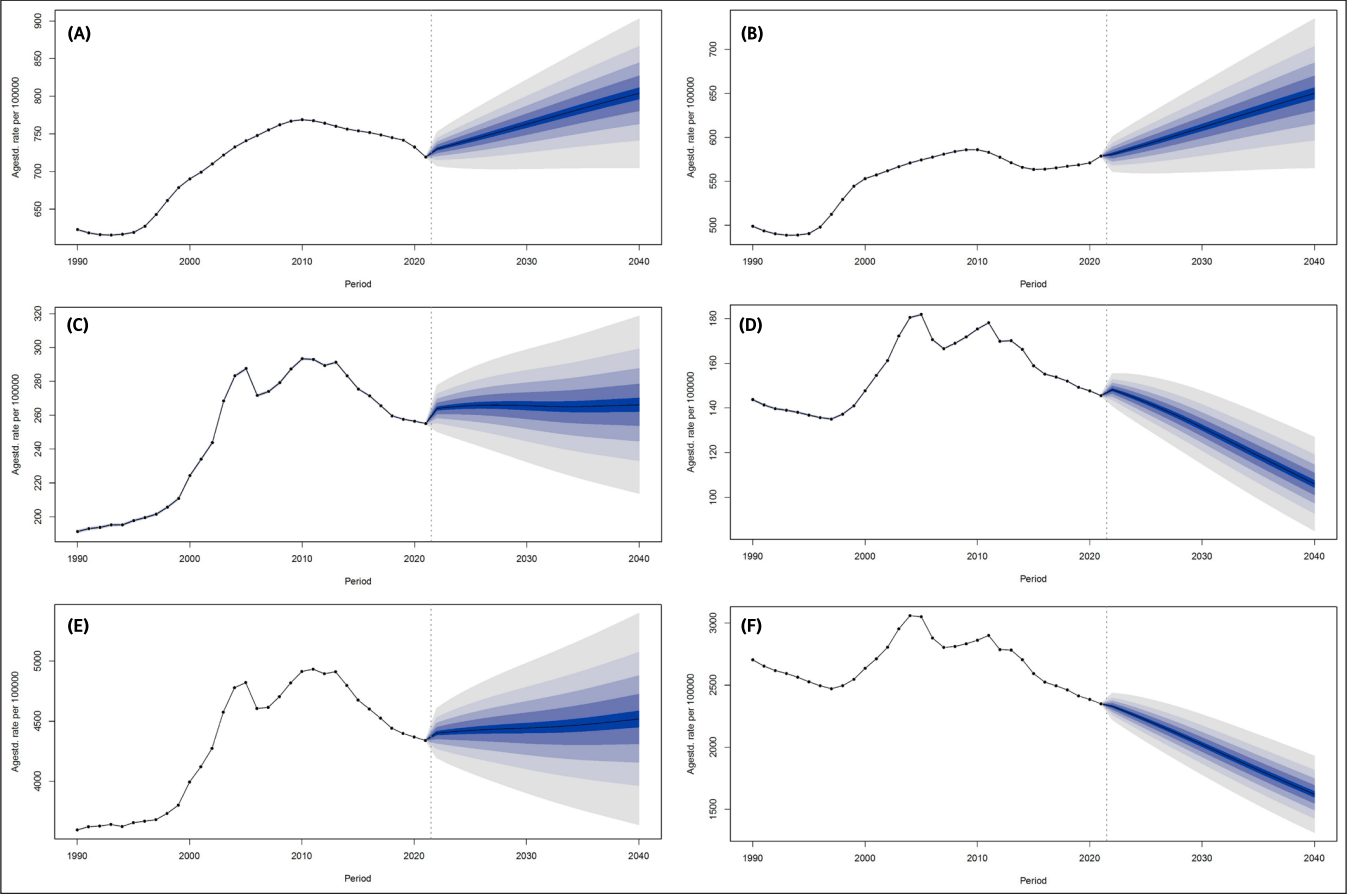
The temporal trends in the age-standardized rates for incidence, mortality, and DALY of ischemic heart disease from 1990–2021 and projections from 2022–2040 in China, stratified by gender. **(A)** The temporal trends in the age-standardized incidence rate of ischemic heart disease from 1990–2021 and projections from 2022 to 2040 in males. **(B)** The temporal trends in the age-standardized incidence rate of ischemic heart disease from 1990–2021 and projections from 2022 to 2040 in females. **(C)** The temporal trends in the age-standardized mortality rate of ischemic heart disease from 1990–2021 and projections from 2022 to 2040 in males. **(D)** The temporal trends in the age-standardized mortality rate of ischemic heart disease from 1990–2021 and projections from 2022 to 2040 in females. (E) The temporal trends in the age-standardized DALY rate of ischemic heart disease from 1990–2021 and projections from 2022–2040 in males. **(F)** The temporal trends in the age-standardized DALY rate of ischemic heart disease from 1990–2021 and projections from 2022–2040 in females. DALY: Disability-adjusted life years.

We used the BAPC model to fit the ASIR, ASMR and ASDR of IHD stratified by sex from 1990–2021 and to predict them in 2040. By 2040, the ASIR in men will increase to 803.86 (95% CI: 685.90–921.81) per 100,000 population with an EAPC of 0.73 (95% CI: 0.56–0.91) (Table 5, Figure 3A). The ASIR for women will reach 650.05 (95% CI: 549.00–751.11) per 100,000 population, which means a slight increase with an EAPC of 0.57 (95% CI: 0.42–0.72) (Table 5, Figure 3B). Men are expected to experience a steeper increase in ASMR than women over the period 2022–2040, with EAPCs of 1.41 (95% CI: 1.05–1.77) and 0.51 (95% CI: 0.16–0.85), respectively (Table 5, Figure 3C, D). Meanwhile, the rate of increase in the ASDR will be higher for men with an EAPC of 0.99 (95% CI: 0.70–1.27) than for women with an EAPC of –0.09 (95% CI: –0.37–0.19) (Table 5, Figure 3E, F).

**Table 5:** Predictions of incidence, mortality, and Disability-adjusted life years of ischemic heart disease in China stratified by gender from 2022 to 2040.


YEAR	AGE-STANDARDIZED INCIDENCE RATE, PER 100 000 (95% CI)		AGE-STANDARDIZED MORTALITY RATE, PER 100 000 (95% CI)		AGE-STANDARDIZED DALY RATE, PER 100 000 (95% CI)	
		
	MALE	FEMALE	MALE	FEMALE	MALE	FEMALE

2022	729.96 (703.03, 756.89)	580.78 (557.20, 604.37)	263.99 (247.71, 280.28)	148.22 (139.55, 156.88)	4400.65 (4154.59, 4646.70)	2329.64 (2202.32, 2456.96)

2023	734.00 (700.31, 767.69)	584.44 (555.32, 613.55)	264.71 (244.71, 284.72)	146.49 (135.78, 157.20)	4409.27 (4098.06, 4720.49)	2294.27 (2135.69, 2452.84)

2024	738.03 (698.32, 777.74)	588.11 (554.01, 622.22)	265.26 (241.94, 288.58)	144.62 (132.19, 157.05)	4416.53 (4048.53, 4784.53)	2257.58 (2073.20, 2441.95)

2025	742.03 (696.74, 787.33)	591.81 (553.04, 630.58)	265.65 (239.28, 292.02)	142.62 (128.69, 156.54)	4422.68 (4002.87, 4842.49)	2219.80 (2013.16, 2426.44)

2026	746.10 (695.49, 796.72)	595.59 (552.35, 638.83)	265.88 (236.65, 295.11)	140.52 (125.26, 155.78)	4427.97 (3959.62, 4896.32)	2181.36 (1955.01, 2407.70)

2027	750.27 (694.51, 806.03)	599.46 (551.89, 647.04)	265.98 (234.01, 297.93)	138.35 (121.88, 154.82)	4432.54 (3917.90, 4947.17)	2142.37 (1898.33, 2386.42)

2028	754.48 (693.71, 815.25)	603.38 (551.56, 655.20)	265.94 (231.37, 300.52)	136.08 (118.52, 153.65)	4436.44 (3877.34, 4995.54)	2102.78 (1842.76, 2362.79)

2029	758.68 (693.00, 824.35)	607.30 (551.30, 663.30)	265.80 (228.72, 302.89)	133.72 (115.16, 152.27)	4439.75 (3837.66, 5041.84)	2062.46 (1788.02, 2336.90)

2030	762.85 (692.33, 833.36)	611.22 (551.09, 671.34)	265.61 (226.09, 305.14)	131.27 (111.81, 150.72)	4442.98 (3798.98, 5086.97)	2021.63 (1734.12, 2309.13)

2031	767.04 (691.73, 842.34)	615.18 (550.95, 679.40)	265.42 (223.51, 307.33)	128.77 (108.50, 149.04)	4446.65 (3761.49, 5131.82)	1980.69 (1681.28, 2280.09)

2032	771.28 (691.20, 851.35)	619.17 (550.86, 687.49)	265.24 (220.98, 309.49)	126.25 (105.23, 147.27)	4451.06 (3725.17, 5176.96)	1939.75 (1629.48, 2250.01)

2033	775.51 (690.69, 860.34)	623.16 (550.76, 695.56)	265.11 (218.54, 311.68)	123.71 (102.01, 145.41)	4456.39 (3690.11, 5222.66)	1898.92 (1578.78, 2219.06)

2034	779.72 (690.16, 869.28)	627.11 (550.64, 703.58)	265.06 (216.20, 313.93)	121.16 (98.85, 143.48)	4462.64 (3656.24, 5269.05)	1858.20 (1529.11, 2187.29)

2035	783.87 (689.59, 878.15)	631.03 (550.48, 711.57)	265.14 (213.98, 316.29)	118.62 (95.74, 141.50)	4470.05 (3623.56, 5316.53)	1817.74 (1480.55, 2154.94)

2036	787.98 (688.98, 886.98)	634.93 (550.31, 719.55)	265.31 (211.87, 318.76)	116.10 (92.71, 139.49)	4478.65 (3591.98, 5365.32)	1777.79 (1433.23, 2122.35)

2037	792.07 (688.34, 895.80)	638.81 (550.10, 727.53)	265.53 (209.80, 321.26)	113.59 (89.74, 137.44)	4488.10 (3561.03, 5415.17)	1738.20 (1386.98, 2089.43)

2038	796.08 (687.62, 904.55)	642.64 (549.82, 735.46)	265.74 (207.74, 323.73)	111.07 (86.82, 135.33)	4497.98 (3530.39, 5465.58)	1698.95 (1341.77, 2056.14)

2039	800.02 (686.81, 913.23)	646.39 (549.45, 743.32)	265.93 (205.68, 326.18)	108.55 (83.94, 133.16)	4508.04 (3499.78, 5516.29)	1659.95 (1297.51, 2022.40)

2040	803.86 (685.90, 921.81)	650.05 (549.00, 751.11)	266.17 (203.67, 328.67)	106.03 (81.11, 130.96)	4518.48 (3469.27, 5567.69)	1621.28 (1254.21, 1988.35)

2022–2040 EAPC (95% CI)	0.73 (0.56, 0.91)	0.57 (0.42, 0.72)	1.41 (1.05, 1.77)	0.51 (0.16, 0.85)	0.99 (0.70, 1.27)	–0.09 (–0.37, 0.19)


EAPC: Estimated annual percentage change, CI: Confidence interval, DALY: Disability-adjusted life years.

## Discussion

IHD is emerging as a major global health challenge, characterised by increasing incidence and mortality. The GBD study allows us to examine the burden of disease, predict and compare disease trends (16). In our study, we first examined and compared the trend of IHD in incidence, prevalence, mortality and DALYs in China and the G20 countries, taking into account different age groups and sexes. We have also made projections for the next two decades. These findings have the potential to inform resource allocation strategies and the development of effective interventions. We found that the decline in ASIR and ASPR over the past 30 years occurred in most G20 countries, which is consistent with previous studies (17,18). From 1990–2021, the incidence, prevalence, mortality and DALYs of IHD increased in China. In fact, the estimated number of incident and prevalent IHD cases in 2021 was 7304573.22 and 63331311.50, accounting for 22.92% and 24.91% of all IHD cases worldwide, respectively. Compared with global averages and other members of the G20, the ASIR and ASPR of IHD in China increased significantly over the study period, with an EAPC of 0.66 (95% CI: 0.50–0.82) and 0.64 (95% CI: 0.56–0.72), respectively. The reasons for this increase in IHD are unclear. One possible hypothesis is that early life exposure to established risk factors for cardiovascular disease, including a westernised diet, high body mass index (BMI) and physical inactivity may lead to genetic and epigenetic changes in lipid modification, inflammation and vasomotor dysfunction (19,20). Our results showed that the proportion of IHD mortality attributable to dietary risk factors in China was 44.26% in 2021, so the increase in incidence rate is mainly due to increased exposure to risk factors. We also found that air pollution and tobacco use were the top five risk factors for IHD in 2021. Air pollution and smoking have been associated with an increased risk of IHD in a dose-dependent manner in many epidemiological studies (21).

High systolic blood pressure and dietary factors are the major risk factors for IHD in China. Accordingly, the Chinese government and public health institutions have issued a series of control and prevention strategies and policies, such as Healthy China 2030, which aims to adopt the most relevant health policies or strategies to improve the overall health of the Chinese population between 2016–2030 (22). The Chinese Centre for Disease Control and Prevention (China CDC) has run several campaigns on non-communicable diseases, such as promoting salt reduction, weight loss and increased physical activity. Air pollution is the third leading cause of death from IHD in China. Tackling air pollution has become a priority for the Chinese government. The State Council issued a National Action Plan for Air Pollution Prevention and Control in 2013, identifying ten detailed strategies and measures for implementation, involving all relevant government agencies (23). Although some progress has been made in China, IHD mortality rates are still increasing, suggesting that there is room for Chinese policymakers to improve IHD prevention and reduce the burden of disease.

In addition, age differences have a significant impact on the burden of IHD. In our study, the age-specific rates of incidence, prevalence, mortality and DALYs gradually increased with age. The high burden of IHD in the elderly may be related to their physical health, related chronic diseases and poor immunity. Therefore, it is advisable to prioritise individuals aged ≥65 years as a key population group for the prevention, early detection and management of IHD.

From 1990 to 2021, the trends in ASMR and ASDR due to IHD showed similar patterns to those of ASIR and ASPR in the G20 countries. In contrast to the increasing trends of ASMR and ASDR in China, ASMR and ASDR due to IHD decreased or increased at a relatively low rate in most G20 countries. This is in line with a recent study which found that mortality rates from IHD showed a downward trend in high SDI countries, particularly Australia, France, Germany, Japan and the United Kingdom (24). Continued progress in early detection and effective treatment of IHD may explain the improvement in ASMR and ASDR. China has initiated some population-based cardiovascular disease screening programmes, such as a large-scale screening of high-risk populations in the Kailuan Cohort Study, which started in 2006, and the China-PAR project, both of which are effectively curbing the significant increasing trend of atherosclerotic cardiovascular disease in China (25,26).

However, according to the forecast results of the BAPC model, the ASIR, ASMR and ASDR of IHD in China will continue to increase over the next 20 years. In addition, men will experience higher levels of ASIR, ASMR and ASDR and will have a more pronounced increasing trend in the rates compared to women. Therefore, promising approaches to optimise prevention and early detection of IHD, including screening of high-risk populations such as young adults with a family history of IHD in first-degree relatives, should be emphasized.

Our study used the latest data from the GBD study to analyse the burden of IHD in China and other G20 countries, comparing it by sex and region. This analysis provides valuable data references for IHD prevention and control efforts. However, the study has several limitations. First, the data provided in GBD 2021 were based on estimates and mathematical modelling, which may affect the accuracy and reliability of the burden estimates. Second, several types of IHD, such as acute myocardial infarction, chronic stable angina, chronic IHD and heart failure were not available in the GBD database for the analysis of IHD burden by subtype. Third, our analysis of the burden of IHD is at a regional and national level, without further analysis of the complex interplay between genetic and environmental factors contributing to the development of IHD.

## Conclusion

This study provides a comprehensive and in-depth analysis of the burden and long-term trends of IHD in China and other G20 countries. The ASIR, ASPR, ASMR and ASDR of IHD increased in China from 1990–2021. There was considerable variation in these rates among the G20 countries. High systolic blood pressure, dietary risks, air pollution, high LDL cholesterol and tobacco were the leading risk factors for IHD mortality in 2021. The burden of IHD in China is projected to increase continuously over the next 20 years.

## Data Accessibility Statement

The original contributions presented in this study are included in the article/supplementary material, further inquiries can be directed to the corresponding authors.

## Additional File

The additional file for this article can be found as follows:

10.5334/gh.1424.s1 Supplementary Figure 1. The age-standardized rates of prevalent cases (A), incidence (B), mortality (C), and DALYs (D) trends due to ischemic heart disease in China and G20 countries from 1990–2021. DALYs: disability-adjusted life-years; G20: group of twenty.
