# Supplementary figures and images for: Trends and Projections of Burden of Ischemic Heart Disease in China Versus Other G20 Countries: A Comparative Study Based on the 2021 Global Burden of Disease Database
Source: Glob Heart. 2025 Apr 3;20(1):37. doi: 10.5334/gh.1424 (PMC11967484; doi:10.5334/gh.1424)

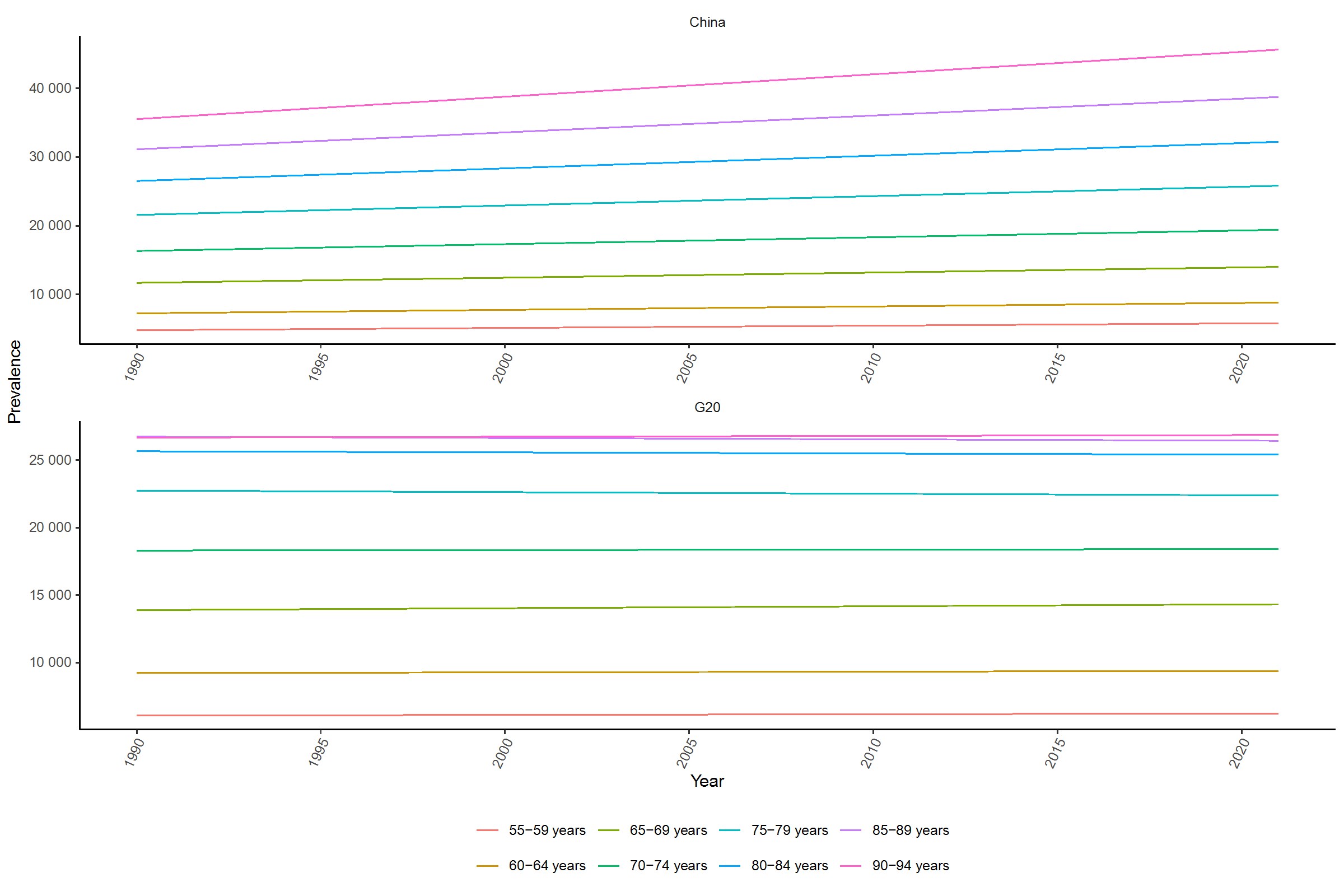

Supplement: Supplementary Figure 1. — The age-standardized rates of prevalent cases (A), incidence (B), mortality (C), and DALYs (D) trends due to ischemic heart disease in China and G20 countries from 1990–2021. DALYs: disability-adjusted life-years; G20: group of twenty. [file gh-20-1-1424-s1.zip › Supplementary Figure 1A-D/Supplementary Figure 1A-D/Supplementary Figure 1A.jpeg]

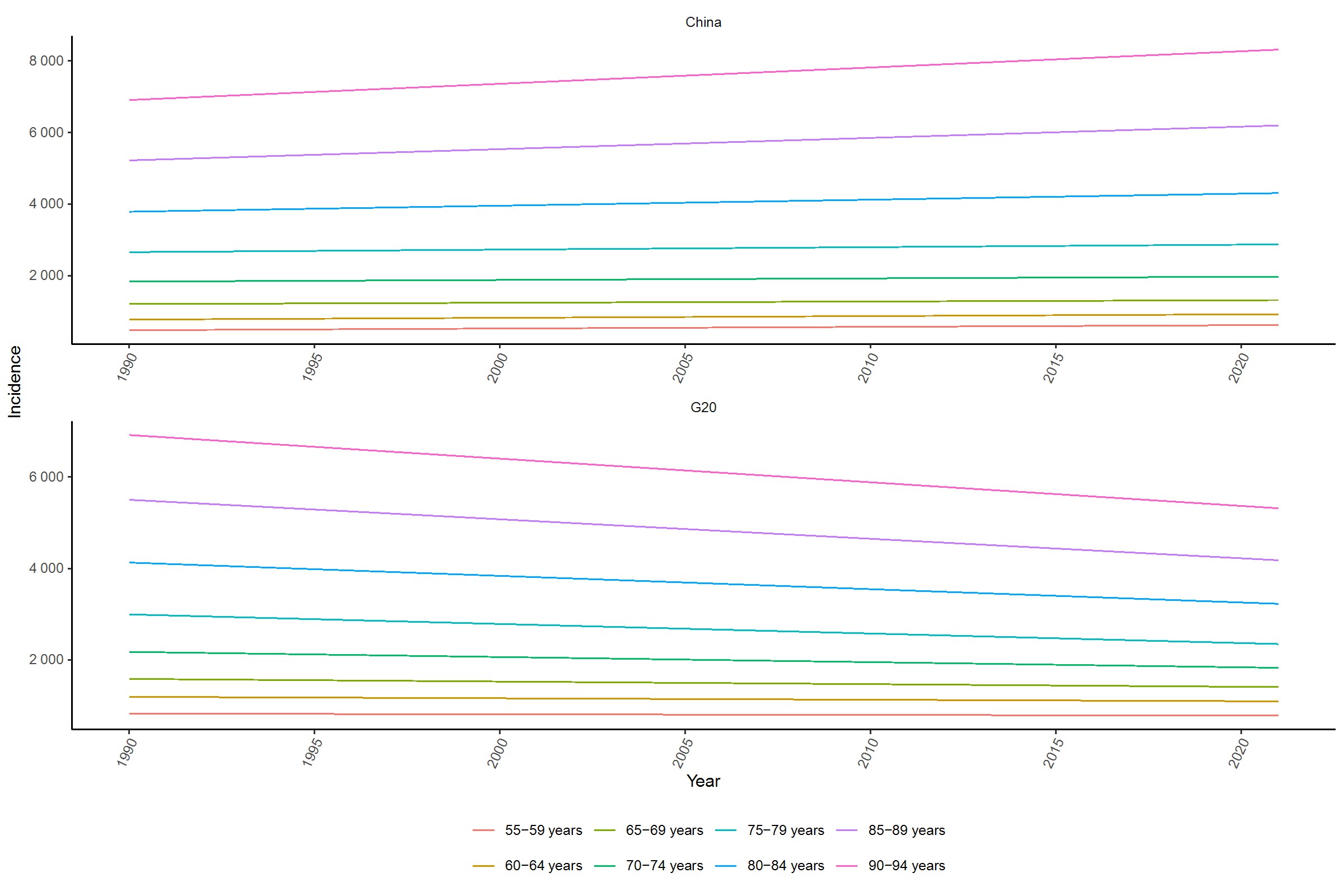

Supplement: Supplementary Figure 1. — The age-standardized rates of prevalent cases (A), incidence (B), mortality (C), and DALYs (D) trends due to ischemic heart disease in China and G20 countries from 1990–2021. DALYs: disability-adjusted life-years; G20: group of twenty. [file gh-20-1-1424-s1.zip › Supplementary Figure 1A-D/Supplementary Figure 1A-D/Supplementary Figure 1B.jpeg]

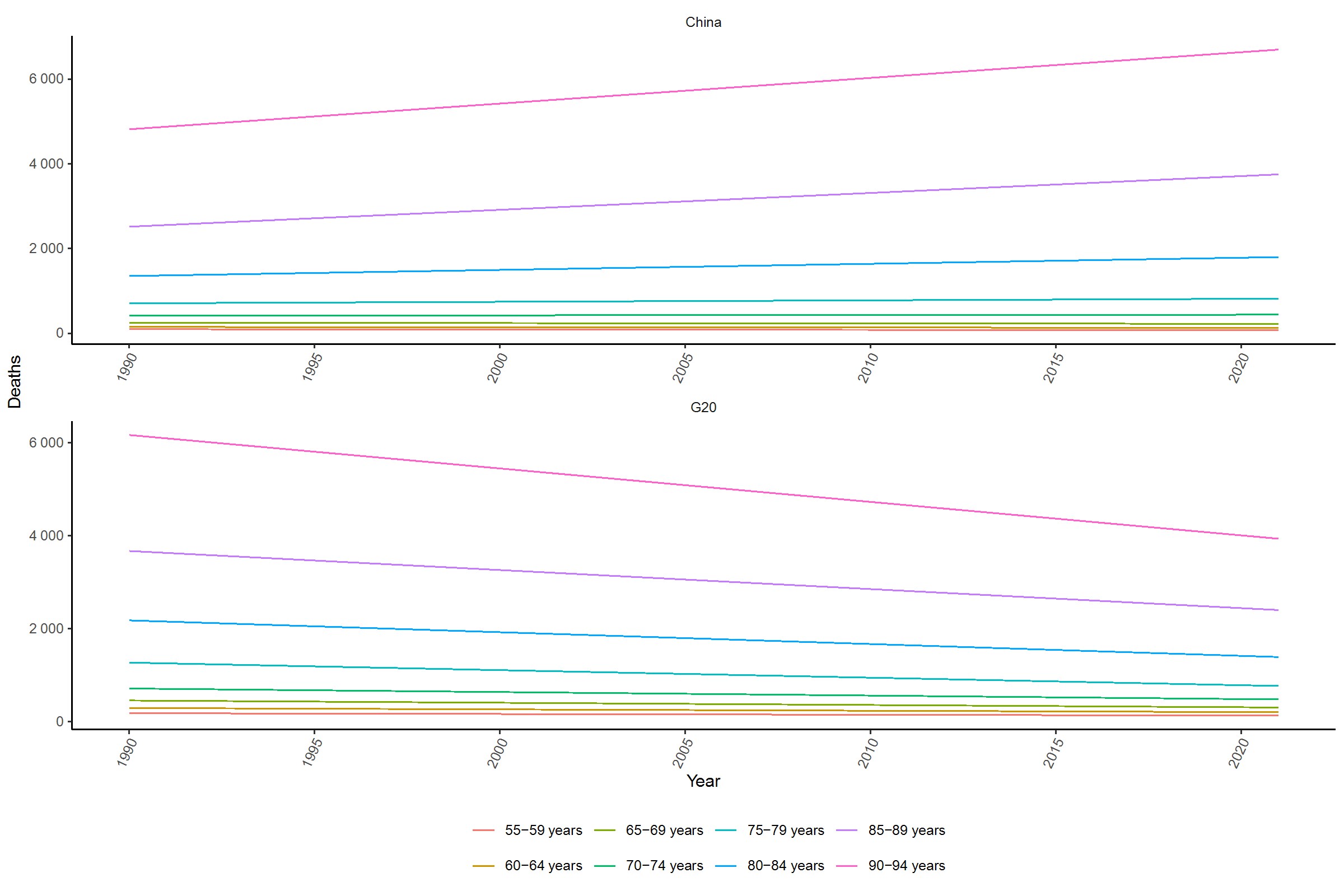

Supplement: Supplementary Figure 1. — The age-standardized rates of prevalent cases (A), incidence (B), mortality (C), and DALYs (D) trends due to ischemic heart disease in China and G20 countries from 1990–2021. DALYs: disability-adjusted life-years; G20: group of twenty. [file gh-20-1-1424-s1.zip › Supplementary Figure 1A-D/Supplementary Figure 1A-D/Supplementary Figure 1C.jpeg]

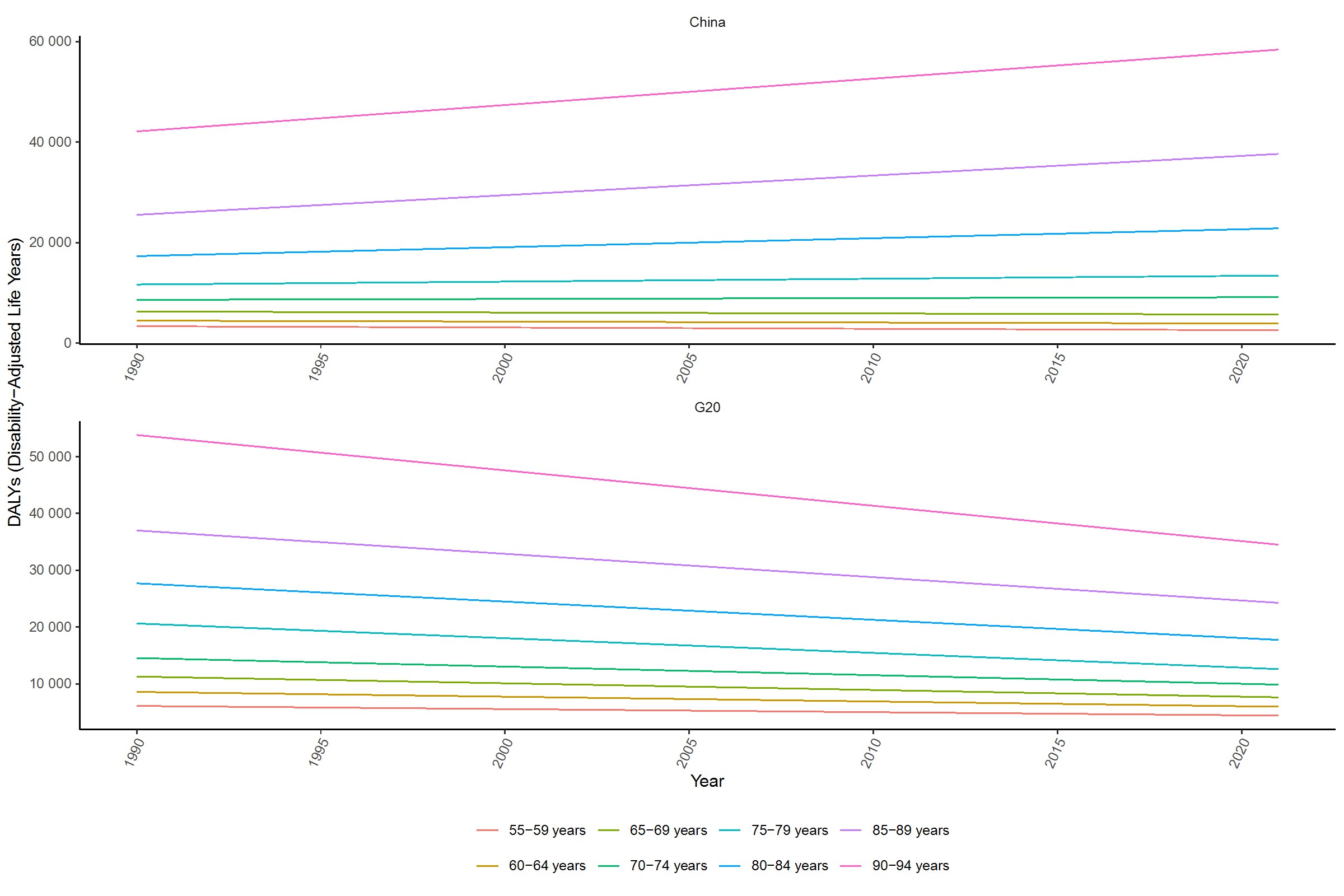

Supplement: Supplementary Figure 1. — The age-standardized rates of prevalent cases (A), incidence (B), mortality (C), and DALYs (D) trends due to ischemic heart disease in China and G20 countries from 1990–2021. DALYs: disability-adjusted life-years; G20: group of twenty. [file gh-20-1-1424-s1.zip › Supplementary Figure 1A-D/Supplementary Figure 1A-D/Supplementary Figure 1D.jpeg]
